# The ribosomal protein S6 in renal cell carcinoma: functional relevance and potential as biomarker

**DOI:** 10.18632/oncotarget.6225

**Published:** 2015-10-25

**Authors:** Maximilian Knoll, Stephan Macher-Goeppinger, Jürgen Kopitz, Stefan Duensing, Sascha Pahernik, Markus Hohenfellner, Peter Schirmacher, Wilfried Roth

**Affiliations:** ^1^ Institute of Pathology, University of Heidelberg, Im Neuenheimer Feld, Heidelberg, Germany; ^2^ Molecular Tumor Pathology, German Cancer Research Center, Im Neuenheimer Feld, Heidelberg, Germany; ^3^ Department of Urology, University of Heidelberg, Im Neuenheimer Feld, Heidelberg, Germany; ^4^ Institute of Pathology, University Medical Center Mainz, Langenbeckstrasse, Mainz, Germany

**Keywords:** mTOR, ribosomal protein S6, everolimus, renal cell cancer, biomarkers

## Abstract

Inhibitors of the mTOR pathway, such as everolimus, are promising compounds to treat patients with renal cell carcinomas (RCCs). However, the precise mechanisms of action are far from clear, and biomarkers predicting the response to mTOR inhibitors are still missing. Here, we provide evidence that in RCCs the rpS6 protein is the major mediator of anti-tumoral effects exerted by everolimus. Inhibition of mTOR signaling results in substantially decreased clonogenicity and proliferation of RCC cells, but did not significantly induce apoptosis. Everolimus effectively blocked protein biosynthesis both *in vitro* and in a novel *ex vivo* tissue slice model using fresh vital human RCC tissue. Compared to other components of the mTOR pathway, phosphorylation of rpS6 was most effectively downregulated by everolimus. Importantly, siRNA-mediated downregulation of rpS6, but not of 4ebp1 or p27, abolished the inhibitory effects of everolimus on proliferation and protein synthesis. Moreover, we analyzed the tissue expression of phosphorylated rpS6 (p-rpS6) and non-phosphorylated rpS6 in a large collection of patients with RCCs (n=598 and n=548, respectively). Expression of both proteins qualified as independent negative prognostic markers with a substantially shorter survival of patients with RCCs exhibiting high levels of rpS6 and p-rpS6. Taken together, our functional studies identified rpS6 as a main mediator of the anti-tumoral activity of Everolimus. Therefore, further (pre-)clinical evaluations of rpS6 as a predictive marker for everolimus-based treatment for RCC patients are warranted. Finally, the combined detection of phosphorylated and non-phosphorylated rpS6 could represent a robust prognostic marker to identify patients with high risk RCCs.

## INTRODUCTION

Renal cell carcinoma (RCC) is a prototype of a chemoresistant and radioresistant malignant tumor, and therapeutic possibilities for metastasized disease have been limited until recently. The introduction of tyrosine-kinase inhibitors and mammalian target of rapamycin (mTOR) inhibitors was a major breakthrough in treatment of RCCs, although primary resistance in a subgroup of patients and development of secondary resistance in almost all patients during the course of treatment are still serious shortcomings of this novel targeted therapeutic approaches. The therapeutic inhibition of the mTOR signaling pathway is clinically achieved by analogues of rapamycin, such as Temsirolimus (CCI-779) and Everolimus (RAD001). Temsirolimus is recommended as first line therapy in clear cell carcinoma with poor risk and non-clear cell carcinoma in any risk constellation. Everolimus is recommended for clear-cell carcinoma after VEGF-R based therapies (second and third line therapy) and as third line therapy after two different TKI [1, 2]. Second and third line treatment with mTOR inhibitors leads to partial response in 3-17% and stable disease in 40-70% of patients [3]. The median time to treatment failure for mTOR inhibitors (second line setting) is 2.5 months [4]. In this regard, further progress in the understanding of the molecular mechanisms of mTOR inhibition as well as novel diagnostic tools to identify patients that will respond to targeted therapy are urgently needed.

The mTOR protein complex functions as a serine/threonine kinase that is mainly activated by the phosphatidylinositol 3-kinase (PI3K)/AKT signaling pathway and plays a pivotal role in control of cell cycle, proliferation and cellular survival both in normal renal tubules and in renal cell carcinoma [5] [6] [7]. Downstream mediators of mTOR are the 70 kDa ribosomal protein S6 kinase (p70S6K), the translational repressor 4EBP1, HIF1A, p27, and Bcl-2 [8, 9]. By activating or inhibiting these targets, mTOR regulates protein synthesis, translation, cell cycle, and apoptosis. The serine/threonine kinase p70S6K can regulate protein synthesis and translation by activating the ribosomal protein S6 (rpS6), but has also other downstream effectors, such as eEF2K, eIF4B, and the GSK3-BAD axis which regulates cell survival [10]. However, despite this knowledge the precise contribution of these downstream targets of mTOR to the success or failure of a mTOR inhibitor-based treatment of RCCs is not well understood. Moreover, the identification of the main mediators of the anti-tumor effects of mTOR inhibitors are of great importance regarding the development of diagnostic techniques to predict the response of individual patients to targeted therapy. This would enable physicians to stratify patients for tailored therapy concepts, increase the invidual benefit from treatment, and prevent unwanted side effects in patients who are not responsive to treatment. Therefore, the aim of our study was to identify crucial mediators of mTOR inhibition in both *in vitro* and *ex vivo* models of RCCs. Further, we investigated the potential of mTOR pathway components as prognostic biomarkers in a large collection of patients with RCCs.

## RESULTS

### Characterization of cellular effects of Everolimus

**Figure 1:**
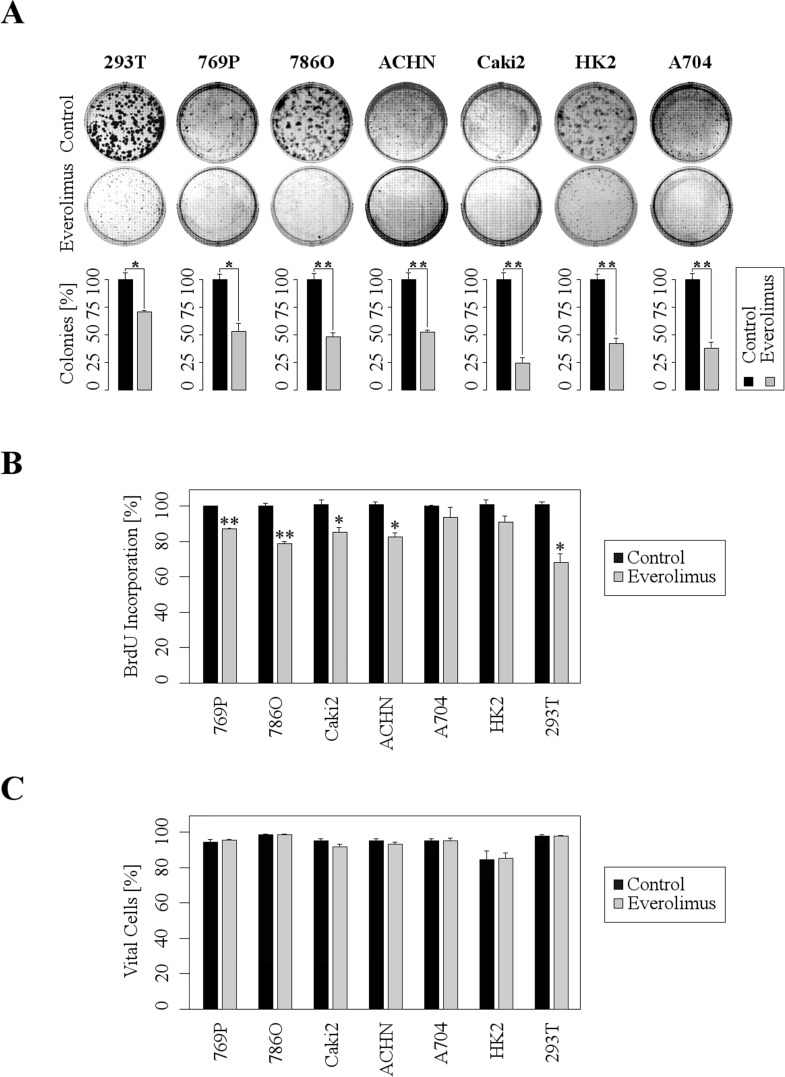
Everolimus leads to inhibition of clonogenicity and proliferation, whereas acute apoptosis is not induced **A.** Upper images show representative wells of 6-well plates, lower bars demonstrate the decrease of colonies after 7 days (ACHN, A704: 14 days) of treatment with 1 μM Everolimus (mean ±SD; *n* = 3, *p < 0.05, ***p < 0.01; *t*-test). **B.** RCC cells were treated with Everolimus (1μM) for 48h and subjected to BrdU assays (mean ±SD; *n* = 3, *p < 0.05, **p < 0.01; *t*-test). **C.** FACS analysis shows no significant difference in cell viability after 48 h of treatment with 1μM Everolimus (mean ±SD; *n* = 3).

In order to functionally investigate the cellular effects of everolimus we examined clonogenicity, proliferation, and viability in a panel of different human RCC cells. Treatment with Everolimus resulted in a strong inhibition of clonogenicity (Figure 1A). In the most sensitive cell line, Caki2, number of colonies were reduced to 25% of control cells. Similarly, Everolimus significantly reduced proliferation in most cell lines except HK2 (which is an immortalized proximal tubule epithelial cell line) and A704 (Figure 1B). Since Everolimus was also reported to induce apoptosis in cancer cells [11, 12], we tested the viability of RCC cells after a 48 h treatment with Everolimus. However, viability was only marginally affected and substantial induction of apoptosis was not observed (Figure 1C). Thus, Everolimus inhibits clonogenicity and proliferation in RCC cells without significant induction of apoptosis.

### Everolimus inhibitis protein synthesis in long-term cultured RCC cells as well as in *ex vivo* tissue samples

**Figure 2:**
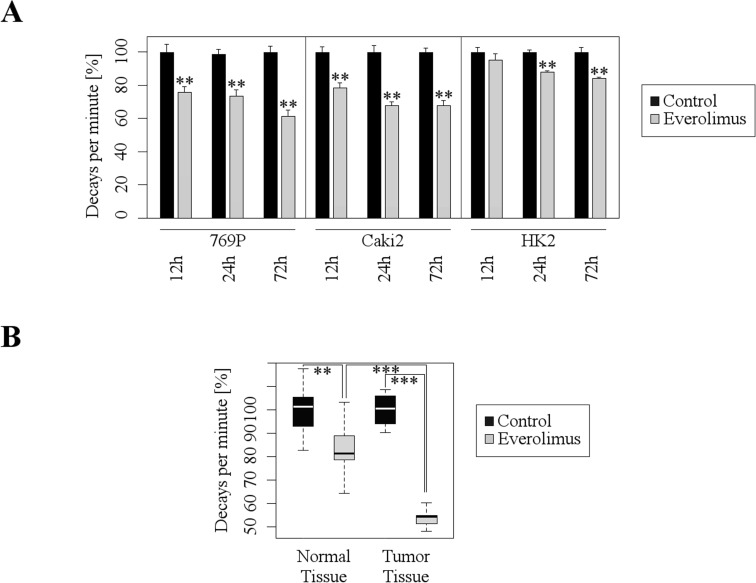
Everolimus inhibits protein synthesis in both cultured RCC cell lines and human tumor tissue **A.** Quantitative analysis of protein synthesis by means of radioactive protein labeling with 35S-methionine after treatment with Everolimus (1 μM) shows significant decrease of protein synthesis (mean ±SD; *n* = 3, **p < 0.01; *t*-test). **B.** *Ex vivo* tissue slice experiments with 35S-methionine labeling show a significantly greater inhibition of protein synthesis after treatment with Everolimus (10 μM) in tumor tissue than in normal tissue (median and 1.+3. quartil; nTumor = 7, nControl = 5; **p < 0.01, ***p < 0.001; *t*-test).

Since mTOR signaling plays a central role for biosynthesis of proteins we examined to which extent Everolimus affects protein synthesis in RCC cells by a ^35^S-methionine protein labelling assay. Everolimus treatment induced a strong inhibition of protein synthesis in 796P and Caki2 RCC cell lines, whereas the inhibitory effect was minor in HK2 cells (Figure 2A). Next, we extended our *in vitro* monolayer cell culture studies by a 3D tumor model accounting for the *in vivo* complexity of human RCC tissue. To this end, we generated 300 μm thick slices from vital, fresh tumor tissue of RCC patients and incubated the tissue with Everolimus. In accordance with the *in vitro* data, Everolimus strongly inhibited protein synthesis in the tumor tissue (Figure 2B). In contrast, Everolimus-dependent inhibition of protein synthesis was only moderate in the corresponding normal tissue. Thus, Everolimus substantially blocked synthesis of proteins in RCC both *in vitro* and *ex vivo*, providing a possible explanation for the inhibitory effects on proliferation and clonogenicity.

### Expression of mTOR pathway components in RCC cell lines and modification by Everolimus

**Figure 3:**
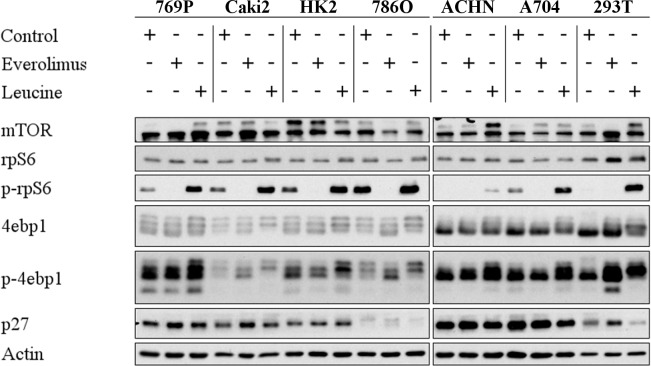
Everolimus leads to dephosphorylation of mTOR targets Immunoblot analysis of whole RCC cell lysates after treatment with Everolimus (1 μM, 72h) or Leucine (10mM, 2h). 20 μg protein were loaded per lane, and Actin was used as a loading control.

In order to functionally investigate the mTOR pathway we characterized the expression of mTOR pathway components in several RCC cell lines by immunoblot analysis (Figure 3). mTOR and rpS6 were expressed in all cell lines tested, and treatment of cells with the mTOR inhibitor Everolimus did not substantially alter the overall expression levels. In contrast, the phosphorylation of rpS6 (p-rpS6) was completely blocked by Everolimus. As a control, cells were treated with leucine which acts as an activator of mTOR by Rheb-dependent stimulation of the mTORC1 kinase activity [13]. Total protein levels of 4ebp1, a translational repressor, were not significantly altered by Everolimus or leucine. However, phosphorylation of 4ebp1 (p-4ebp1) was increased upon leucin treatment. Everolimus treatment resulted in a non-consistent moderate up- or down-regulation of p-4ebp1. No significant regulation was observed for p27 after treatment with Everolimus or leucine. These results indicated that mTOR signaling was intact in the cell lines tested, and that rpS6 was the most significantly and consistently regulated target influenced by Everolimus.

### rpS6 mediates the effects of Everolimus on proliferation and protein synthesis

**Figure 4:**
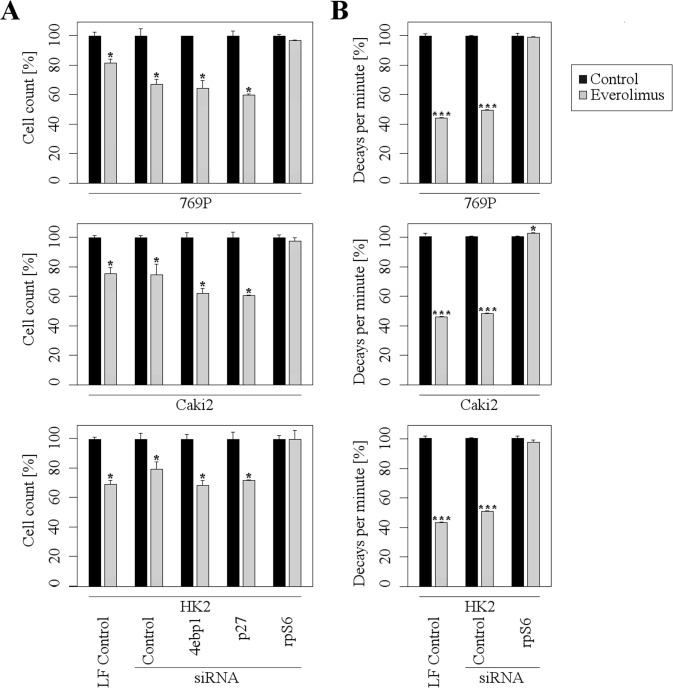
rpS6 mediates the inhibitory effects of Everolimus A further inhibition of Everolimus (1μM) on proliferation **A.** and protein synthesis **B.** is abolished in rpS6 depleted cells, but not in 4ebp1 or p27 depleted cells. Note that values are normalized to 100% to allow a direct comparison between different siRNAs. LF = Lipofectamine (mean ±SD; *n* = 3, *p < 0.05, **p < 0.01; *t*-test). Protein synthesis was measured after 35S-methionine labeling.

As a next step, we investigated which of the mTOR downstream targets is most important for the mediation of the inhibitory Everolimus effects. Therefore, we downregulated rpS6, p27, and 4ebp1 by an siRNA approach and subsequently treated the RCC cells with Everolimus. Importantly, the down-regulation of rpS6 completely abolished a further Everolimus-dependent inhibition of proliferation, whereas p27- and 4ebp1-downregulated cells were still susceptible to the inhibitory effects of Everolimus (Figure 4A). Accordingly, siRNA-mediated suppression of rpS6 blocked a further inhibitory effect of Everolimus on protein synthesis (Figure 4B). These results indicate that rpS6 plays a central role in mediating the inhibitory effects of Everolimus on mTOR signaling in RCC cells.

### rpS6 and p-rpS6 are independent prognostic markers in patients with RCCs

**Figure 5:**
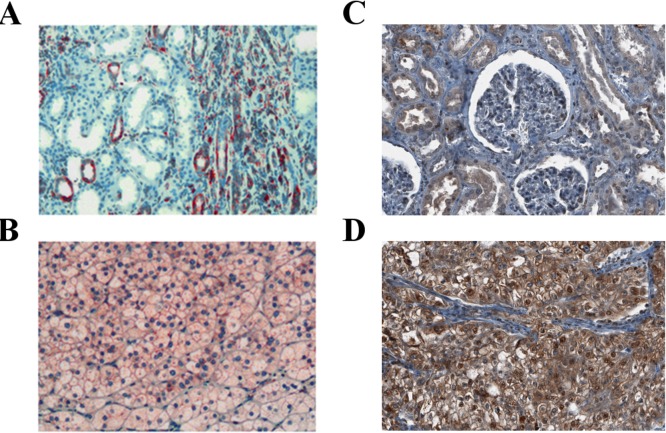
Expression of rpS6 and p-rpS6 in human RCC tissue Expression of rpS6 (red staining) in normal renal tissue **A.** and a clear-cell RCC **B.**, as well as expression of p-rpS6 (brown staining) in normal renal tissue **C.** and a clear-cell RCC **D.** was assessed by immunohistochemistry.

Given the paramount role of mTOR signaling in RCCs and the central functional role of rpS6, we wondered whether the expression of rpS6 and its phosphorylated form in RCCs is associated with clinical or pathological features of the tumors. To this end, we took advantage of a tissue micro array containing tumor tissue and corresponding normal renal tissue samples from 838 patients with RCCs [14, 15]. Expression of rpS6 and p-rpS6 was analyzed by immunohistochemistry. As depicted in Figure 5, rpS6 and p-rpS6 was immunohistochemically detected in the cytoplasm in variable intensity. Altogether, 580and 598 cases were successfully scored for expression of rpS6 and p-rpS6, respectively. The remaining cases with insufficient tumor tissue, fixation artefacts or tumor independent death were excluded from further analyses.

Table 1 provides a summary of the clinical and pathological features. The median follow-up time was 49 months (mean follow-up: 59 months). At the last follow-up, of 776 patients, 208 (27%) had died of RCC and 440 (57%) were still alive.

**Table 1:** Summary of clinical and pathologic features

Feature	n (%) rpS6	n (%) p-rpS6
Sex		
Male	351 (61)	369 (62)
Female	229 (39)	229 (38)
Age at surgery (y)		
< 65	352 (60)	368 (61)
>= 65	228 (40)	230 (39)
Tumor extent (TNM 2009)		
pT1	310 (53)	317 (53)
pT2	62 (11)	71 (12)
pT3a	76 (13)	73 (12)
pT3b	105 (18)	106 (18)
pT3c	16 (3)	20 (3)
pT4	11 (2)	11 (2)
Regional lymph node metastasis (TNM 2009)		
N0/pN0	528 (91)	549 (92)
pN1/pN2	52 (9)	49 (8)
Distant metastasis (TNM 2009)		
M1	114 (20)	112 (19)
M0	466 (80)	486 (81)
Grade of malignancy		
G1	137 (24)	130 (22)
G2	335 (58)	356 (60)
G3	100 (17)	105 (18)
G4	2 (0)	1 (0)
Type of surgery		
Radical nephrectomy	492 (85)	510 (85)
Partial nephrectomy	88 (15)	88 (15)
Histopathological subtype		
Clear cell (conventional) RCC	486 (84)	496 (83)
Papillary (chromophil) RCC	54 (9)	56 (9)
Chromophobe RCC	23 (4)	27 (5)
Spindle cell carcinoma	6 (1)	6 (1)
Collecting duct carcinoma	3 (0)	2 (0)
Unclassified	8 (1)	11 (1)

### rpS6

**Figure 6:**
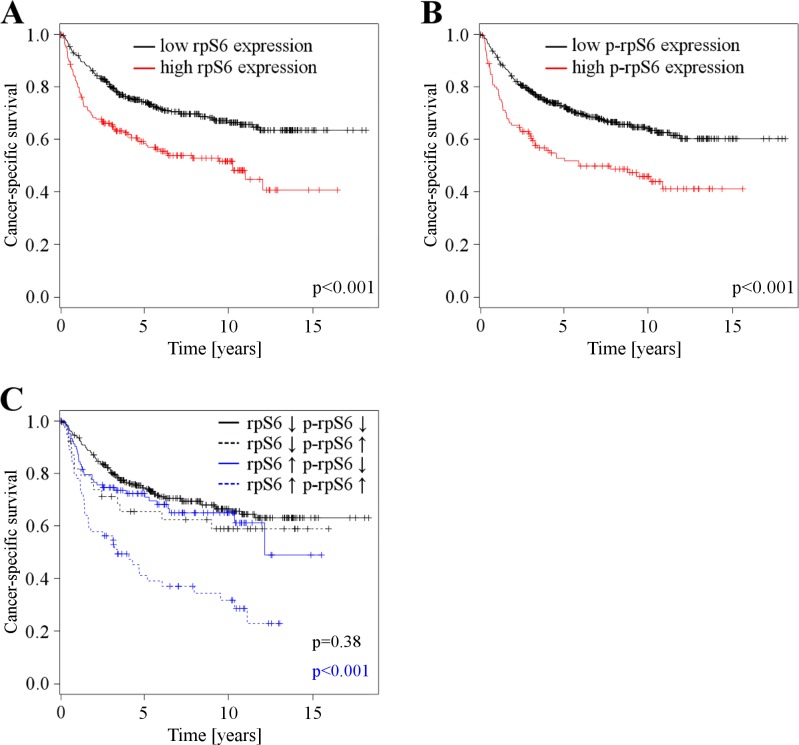
**A.** Cancer specific survival depending on rpS6 expression levels (*n* = 580), patients with low rpS6 expression levels (*n* = 387) *vs*. patients with high rpS6 expression levels (*n* = 193). **B.** Cancer specific survival depending on p-rpS6 expression levels (*n* = 598), patients with low p-rpS6 expression levels (*n* = 473) *vs*. patients with high p-rpS6 expression levels (*n* = 125). **C.** Cancer specific survival depending on p-rpS6 expression in patients with low (*n* = 339) and high rpS6 (*n* = 167) expression. Patients with low rpS6 expressing RCCs are further divided into groups with low (*n* = 300) and high (*n* = 39) p-rpS6 expression, the same partitioning is done for patients with high rpS6 expression: low p-rpS6 (*n* = 103) and high p-rpS6 (*n* = 64).

High rpS6 expression (defined as intensity > = 1) was observed in 193 patients (33 %). Using Fisher's exact tests, high rpS6 expression levels were significantly associated with regional lymph node metastasis, grade of malignancy, partial nephrectomy, and non clear-cell histology (Table 2; for an analysis of localized vs. metastasized cases see Suppl. Table 1 and 2). Univariate survival analyses showed that high rpS6 expression is associated with a poor clinical outcome in patients with RCC (*p* < 0.001, Figure 6A, cancer-specific survival; for an analysis of progression-free survival see Suppl. Figure 1A; for an analysis of only clear-cell RCC see Suppl. Figure 2A). Next, we investigated the impact of rpS6 expression on the RCC related cancer specific survival and progression free survival by multivariate analysis. Multivariate Cox regression analysis included rpS6 expression, Karnofsky performance status, tumor extent, regional lymph node metastasis, distant metastasis, grade of malignancy, type of surgery, gender, and histological subtype (Table 3). RpS6 emerged as a significant prognostic factor in the whole patient group (Table 3, cancer-specific survival: 1.8 [1.4-2.4], *p* < 0.001; Suppl. Table 3, progression-free survival: 1.6 [1.2-2.2], *p* = 0.001) as well as in the group of patients with localized (Suppl. Table 4, cancer-specific survival: 1.6 [1.1-2.5], *p* = 0.028; Suppl. Table 3, progression-free survival: 1.5 [1-2.3], *p* = 0.039) and metastasized disease (Suppl. Table 4, cancer-specific survival: 2.1 [1.4-3.2], *p* = 0.001; Suppl. Table 3, progression-free survival: 2.1 [1.4-3.2], *p* = 0.001). Similar results were obtained for the histological subgroup of clear-cell RCC (Suppl. Tables 5 and 6).

**Table 2:** Comparison of rpS6 / p-rpS6 expression levels and clinical and pathologic features

Feature	rpS6			p-rpS6		
	high 193 (33 %)	low 387 (67 %)	p	high 125 (21 %)	low 473 (79 %)	p
Sex			0.86			0.25
Male	118 (20)	233 (40)		83 (14)	286 (48)	
Female	75 (13)	154 (27)		42 (7)	187 (31)	
Age at surgery (y)			1			0.91
<65	117 (20)	235 (41)		76 (13)	292 (49)	
>=65	76 (13)	152 (26)		49 (8)	181 (30)	
Karnofsky index			0.06			**<0.001**
>=1	74 (13)	117 (20)		60 (10)	145 (24)	
<1	119 (21)	270 (47)		65 (11)	328 (55)	
Tumor extent			0.17			**<0.01**
pT1/2	116 (20)	265 (44)		68 (11)	320 (53)	
pT3/4	77 (13)	131 (23)		57 (10)	153 (26)	
Regional lymph node metastasis			**<0.01**			**<0.001**
N0/pN0	167 (29)	361 (62)		104 (17)	445 (74)	
pN1, pN2	26 (4)	26 (4)		21 (4)	28 (5)	
Distant metastasis			0.08			**0.01**
M1	147 (25)	319 (55)		92 (15)	394 (66)	
M0	46 (8)	68 (12)		33 (6)	79 (13)	
Grade of malignancy			**<0.01**			**<0.001**
G1/2	146 (25)	332 (57)		88 (15)	404 (68)	
G3/4	47 (8)	55 (9)		37 (6)	69 (12)	
Type of surgery			**0.01**			0.57
Radical nephrectomy	153 (26)	339 (58)		109 (18)	401 (67)	
Partial nephrectomy	40 (7)	48 (8)		16 (3)	72 (12)	
Histopathologic subtype#			**0.006**			0.68
Clear cell (conventional) RCC	150 (26)	336 (58)		102 (17)	394 (66)	
Papillary (chromophil) RCC	27 (5)	27 (5)		16 (3)	40 (7)	
Chromophobe RCC	4 (2)	19 (3)		3 (1)	24 (4)	
Spindle cell carcinoma	5 (2)	1 (0)		0 (0)	6 (1)	
Collecting duct carcinoma	3 (2)	0 (0)		2 (0)	0 (0)	
Unclassified	4 (2)	4 (2)		2 (0)	9 (1)	

# clear cell vs. other subtypes

**Table 3:** Uni- and multivariate analyses of rpS6 and p-rpS6 expression and clinical/pathologic features for the prediction of cancer specific survival in patients with RCCs

	Univariate Analysis		Multivariate Analysis	
	Hazard Ratio (95 % CI)	p	Hazard Ratio (95 % CI)	p
**rpS6 & p-rpS6**	1.7 (1.3-2.3)	**<0.001**	1.9 (1.4-2.6)	**<0.001**
Karnofsky*	2.6 (1.9-3.4)	**<0.001**	1.6 (1.2-2.2)	**0.002**
Tumor extent*^◦^*	5.7 (4.2-7.8)	**<0.001**	2.9 (2-4.2)	**<0.001**
Regional lymph node metastasis*^•^*	7.1 (4.9-10.1)	**<0.001**	1.6 (1.1-2.4)	**0.03**
Distant metastasis*^±^*	10.6 (7.7-14.4)	**<0.001**	5.1 (3.5-7.3)	**<0.001**
Grade of malignancy*^⊗^*	5.4 (3.9-7.3)	**<0.001**	1.8 (1.3-2.5)	**0.001**
Type of surgery^t^	4.5 (2.1-9.7)	**<0.001**	1.9 (0.9-4.3)	0.10
Sex ^=^	1.6 (1.2-2.2)	**0.003**	1.3 (0.9-1.8)	0.15
Histopathologic subtype*^<^*	1.6 (1.2-2.2)	**0.003**	1.2 (0.7-1.9)	0.5
**p-rpS6 Expression*∗***	1.9 (1.4-2.6)	**<0.001**	1.4 (1-1.9)	**0.03**
Karnofsky*	2.3 (1.8-3)	**<0.001**	1.7 (1.3-2.2)	**<0.001**
Tumor extent*^◦^*	5.6 (4.2-7.5)	**<0.001**	2.8 (2-3.8)	**<0.001**
Regional lymph node metastasis*^•^*	6.8 (4.9-9.5)	**<0.001**	1.7 (1.2-2.5)	**0.006**
Distant metastasis*^±^*	10.8 (8.1-14.4)	**<0.001**	5 (3.6-7)	**<0.001**
Grade of malignancy*^⊗^*	5.3 (4-7)	**<0.001**	1.9 (1.4-2.6)	**<0.001**
Type of surgery^t^	3.4 (1.9-6)	**<0.001**	1.2 (0.7-2.3)	0.51
Sex ^=^	1.6 (1.2-2.2)	**0.001**	1.4 (1-1.9)	**0.04**
Histopathologic subtype*^<^*	1.6 (1.2-2.2)	**0.001**	1.1 (0.7-1.8)	0.56
**rpS6 Expression*∗***	1.8 (1.4-2.4)	**<0.001**	1.8 (1.4-2.4)	**<0.001**
Karnofsky*	2.5 (1.9-3.2)	**<0.001**	1.7 (1.3-2.3)	**<0.001**
Tumor extent*^◦^*	5.9 (4.3-7.9)	**<0.001**	2.8 (2-3.9)	**<0.001**
Regional lymph node metastasis*^•^*	5.9 (4.2-8.3)	**<0.001**	1.4 (0.9-2)	0.13
Distant metastasis*^±^*	10.3 (7.7-13.8)	**<0.001**	4.9 (3.5-6.8)	**<0.001**
Grade of malignancy*^⊗^*	5.8 (4.3-7.7)	**<0.001**	1.9 (1.4-2.6)	**<0.001**
Type of surgery^t^	5 (2.4-10.1)	**<0.001**	1.9 (0.9-3.9)	0.1
Sex ^=^	1.6 (1.2-2.2)	**0.001**	1.5 (1.1-2)	0.015
Histopathologic subtype*^<^*	1.6 (1.2-2.2)	**0.001**	1.1 (0.7-1.8)	0.6

∗ Immunistochemical intensity low vs. high

* <80% vs >= 80%

◦ pT3/pT4 vs pT1/pT2

• pN1/pN2 vs pN0/N0

± M0 vs M1

⊗ G1/G2 vs G3/G4

= Male vs female

t Radical vs partial nephrectomy

< Clear-cell (conventional) vs other types

### p-rpS6

Elevated p-rpS6 expression levels were observed in the tumor tissue of 125 patients (21%). High p-rpS6 expression levels were significantly associated with tumor extent, regional lymph node metastasis, distant metastasis, and grade of malignancy (Table 2; for an analysis of localized vs. metastasized cases see Suppl. Table 1 and 2). Univariate survival analyses showed that high rpS6 expression is associated with a poor clinical outcome in patients with RCC (*p* < 0.001, Figure 6B, cancer-specific survival; for an analysis of progression-free survival see Suppl. Figure 1B; for an analysis of only clear-cell RCC see Suppl. Figure 2B). Multivariate Cox analysis identified p-rpS6 as a significant prognostic factor in the whole patient group (Table 3, cancer-specific survival: 1.4 [1-1.9], *p* = 0.03) and patients with metastatic disease (Suppl. Table 4, cancer-specific survival: 1.6 [1-2.5], *p* = 0.03; Suppl. Table 3, progression-free survival: 1.6 [1-2.5], *p* = 0.03). Similar results were obtained for the histological subgroup of clear-cell RCC (Suppl. Tables 5 and 6).

In patients with RCC expressing both high rpS6 and p-rpS6 levels, survival times were even shorter (cancer-specific survival: Figure 6C; for an analysis of progression-free survival see Suppl. Figure 1C; for an analysis of only clear-cell RCC see Suppl. Figure 2C). Multivariate analysis identifies the combination of both markers as prognostic relevant in the whole patient group (Table 3, cancer-specific survival: 1.9 [1.4-2.4], *p* < 0.001; Suppl. Table 3, progression-free survival: 1.8 [1.3-2.4], *p* < 0.001), patients with localized (Suppl. Table 4, cancer-specific survival: 1.9 [1.2-2.9], *p* = 0.007; Suppl. Table 3, progression-free survival: 1.7 [1.1-2.6], *p* = 0.013) and patients with metastasized disease (Suppl. Table 4, cancer-specific survival: 2.4 [1.6-3.9], *p* < 0.001; Suppl. Table 3, progression-free survival: 2.4 [16-3.9], *p* < 0.001).

## DISCUSSION

Despite recent advances in systemic therapy of metastasized RCC, most patients are not cured and only 32% of patients treated with Temsirolimus showed clinical benefit, defined as objective response or stable disease > = 24 weeks [16]. Treatment with Everolimus leads to partial response in 14%, and stable disease in 73 % and 57 % after three and six months [17]. The application of targeted therapies is so far not guided by biomarkers. Thus, the development of predictive biomarkers is a priority in translational research in RCC to permit rational tailored treatment of individual patients.

Temsirolimus (CCI-779) and Everolimus (RAD001) are rapamycin derivatives and form a complex with the 12-kDa FK506-binding protein (FKBP12) that inhibits mTOR signaling [18] [19]. Along with several proteins, mTOR forms two distinct complexes, named mTORC1 and mTORC2, whereas mTORC1 but not mTORC2 is inhibited by rapamycin analogs. The mTORC1 pathway regulates major cellular functions, like proliferation or protein synthesis [20]. Downstream targets of mTOR signaling pathways are, among others, p70S6K, HIF1A, and 4E-BP1. The protein kinase p70S6K phosphorylates several downstream substrates, such as rpS6, and thereby promotes protein synthesis [21].

In our study, the mTOR inhibitor Everolimus mainly affected clonogenicity and proliferation of RCC cells, without substantial impact on cell death pathways. Although Everolimus was reported to induce apoptosis in leucemia cells [22], significant triggering of apoptotic cell death was not observed in our RCC model. Since Everolimus universally inhibits mTORC1, the differential apoptosis response might be caused by its varying effects on Akt dependent on cell and tissue type [23]. The major inhibitory effect of Everolimus on protein synthesis was confirmed both *in vitro* and *ex vivo*. Our innovative *ex vivo* tissue slice model allowed us to directly study the effects of Everolimus on human vital RCC tissue while maintaining the original tumor-stroma interaction. In this model, Everolimus substantially inhibited protein synthesis in RCC tissue, whereas the inhibitory effect on non-tumor tissue was much weaker. This finding points to a higher dependency on the mTOR pathway in tumor samples.

One important result of our study is the crucial functional relevance of rpS6 in mediating the anti-tumor effects of Everolimus. The siRNA-mediated downregulation of rpS6, but not of 4ebp1 or p27, abolished the inhibitory effects on proliferation and protein synthesis of Everolimus. In contrast, the functionality of 4ebp1 to repress translation could be taken over by its counterpart 4ebp2 and therefore compensate for its loss. Functional redundancy exists also for the cell cycle inhibitor p27, e.g. by the other Kip/Cip proteins p21 and p57. Although the importance of the protein kinase p70S6K for mTOR-mediated cellular effects is well established, the contribution of the various downstream effectors of p70S6K, such as rpS6 and others, is far from clear. Other effectors modulate protein synthesis (eEF2K, eIF4B, Pdcd4), cytoskeletal rearrangement, proliferation, splicing, cell survival and an mTOR feedback loop [24].

Importantly, the central functional role of rpS6 opens up the possibility to exploit the measurement of rpS6 expression levels as a predictive biomarker that prognosticates whether a tumor will respond to pharmaceutical mTOR inhibition or not. A limited number of studies addressed this question. Iwenofu et al. identified p-rpS6 in a group of 20 patients with high grade metastatic sarcoma as an marker for early clinical response to AP23573 treatment (an mTOR inhibitor). Highly p-rpS6 expressing tumor samples showed stable disease after two cycles of therapy, low expressing tumors showed progressive disease [25]. In RCCs, Cho et al. showed that immunohistochemical detection of p-rpS6 may have predictive potential for treatment with mTOR inhibitors. Tissue samples of 20 patients were stained for p-rpS6 and subsets of them for CA IX, p-Akt and PTEN. Partial or minimal response to Temsirolimus was associated with high p-rpS6 expression (*p* = 0.02) [26]. Li et al. confirmed p-rpS6 as a potential predictive marker (*n* = 18, improved PFS) and identified p-mTOR as an additional marker [27]. However, due to the limited study population results were preliminary and have to be confirmed by further studies. On the other hand, baseline amounts of PTEN and HIF-1α are not usable as predictive markers in Temsirolimus treated RCCs [28].

Since it is difficult to recruit enough eligible patients for a well designed study with sufficient statistical power that addresses the question of predicting mTOR inhibitor efficacy, future multicentric trials will have to validate the predictive power of rpS6 expression in a clinical setting. In any case, the results presented in this paper warrant a further evaluation of rpS6 as a companion diagnostic for the therapeutic use of mTOR inhibitors.

Although the identification of a predictive biomarker for targeted therapies is clinically most urgently required, in the case of RCCs there is also a medical need for reliable prognostic markers that are able to distinguish high risk from low risk patients. Patients who are at high risk for relapse or metastasis could be monitored more closely and offered more aggressive therapies or the inclusion into clinical trials. Therefore, we studied the expression of rpS6 and p-rpS6 in a large collection of RCC patients and compared the expression levels with clinical and pathological features. We found that high expression of rpS6 or p-rpS6 is associated with high grade RCCs. Furthermore, univariate and multivariate survival analyses showed that high expression of either one is associated with a poor clinical outcome in patients. Importantly, the combined assessment of rpS6 and p-rpS6 expression could further improve the prognostic significance. Since the immunohistochemical assessment of protein expression levels for prognostic and/or predictive purposes is already in clinical use in other tumor types (e.g. hormone receptors and proliferative activity in breast cancer), the analysis of rpS6 and p-rpS6 for prognostication of RCC patients seems feasible. Taken together, rpS6 and p-rpS6 represent promising candidates for prognostic and predictive biomarkers in patients with RCCs, and their possible clinical usage should be further validated by future investigations.

## MATERIALS AND METHODS

### Cell culture

Human RCC cell lines 769-P, 786-O, Caki-2, ACHN, A704, 293T and HK-2 were purchased from ATCC (Rockville, MD, USA). These celllines where authenticated by ATCC through short tandem repeat profiling. Cells where cultured for at most 10 passages at 37°C in 5% CO2 atmosphere and maintained in RPMI-1640 medium (Life Technologies, Gaithersburg, MD, USA) with additional 10% fetal calf serum, 1 mM glutamine, 25 mM glucose and 1 % penicillin/streptomycin (Life Technologies, Gaithersburg, MD, USA).

### Transfection

Endogenous protein was specifically knocked down transiently by transfecting cell lines with short interfering RNA (siRNA) oligonucleotides at concentration of 10 nM (p27 and 4ebp1) and 20 nM (rpS6) using Lipofectamine 2000 (Invitrogen, Carlsbad, CA, USA). Three different rpS6 siRNAs were obtained from Dharmacon (Lafayette, CO, USA) and Santa Crus (Dallax, TX, USA): #J-003024-11, #J-003472-07, #sc-36424. Other siRNAs used were obtained from Dharmacon as well: #J-003472-07 against p27 and #J-003005-13 against 4ebp1. Non-specific siRNA was used as a control (#D-001810-10).

### Immunoblot analysis

Ice-cold PBS was used to rinse cells, followed by lysis with lysis buffer (50 mM Tris-HCl (pH 8.0), 120 mM NaCl, 5 mM EDTA, 0,5% Triton X-100) containing PMSF (1 mM), proteinase inhibitors (Roche, Mannheim, Germany, #1697498) and phosphatase inhibitors (10 mM NaPPi, 200 μM NaVO3, 25 mM NaF). Incubation for 15 min on ice was followed by centrifugation of lysates at 16 000 g for 20 min. Bradford Assays (Bio-Rad, Munich, Germany) were used to measure protein concentrations. For electrophoresis, 20-40 μg of protein was separated with 10-15% polyacrylamid gels and blotted onto nitrocellulose membranes (Bio-Rad) by standard procedures. Membranes where washed, incubated over night with primary antibody, washed again and incubated with secondary antibody (1:3000) coupled to horseradish peroxidase (Bio-Rad). Visualization was performed by an enhanced chemiluminescence detection system (GE Healthcare, Munich, Germany). Following primary antibodies were used: anti-beta-Actin (Sigma, Deisenhofen, Clone AC15, A5441), anti-4ebp1 (Cell Signaling, Boston, #9644), anti-p-4ebp1 (Cell Signaling, #2855), anti-rpS6 (Cell Signaling, #2317), anti-p-rpS6 (Cell Signaling, #4858), anti-p27 (DakoCytomation, Clostrup, Clone SX53G8, M 7203). Everolimus was usually used in concentrations of 1 μM for 72 h, leucine (Sigma-Aldrich, St. Louis, MO) for 2 h at concentrations of 10 mM.

### Cytotoxicity assays

10^4^ cells were plated in 96-well plates, adhered for 24 h and treated with Everolimus for 72 h. The surviving ratio was assessed by staining with crystal violet [29]. After removing of the supernatant, the cells were incubated in 2% crystal violet solution in 20% methanol for 10 min. After washing in running-tap water the plates were air-dried for 24 h. The portion of bound crystal violet was solubilized by the addition of a 0.1 M sodium citrate buffer in 50% ethanol. Absorption was measured at 550 nm using a microplate reader (Bio-Rad, Munich, Germany).

### FACS

FACS measurements of propidium iodid dyed cells and their supernatants were performed with a PAS II Flowmeter (Partec, Munich, Germany) to assess the amount of vital cells and their cell cycle distribution. Cells were centrifuged at 1000 rpm for 5 min and Nicoletti agens added (0.1 % Na-Citrat, 0.1 % Triton X-100, pH 7.4). Data was acquired and analysed with the MultiCycle software (Phoenix Flow Systems, San Diego, CA, USA).

### Proliferation assay

Assessment of proliferation was performed in 96-well plates. 10^4^ cells were seeded, adhered for 24 hours and treated with BrdU for 48h (Amersham Cell Proliferation Kit, RPN250, GE Healthcare, Solingen). Incubation with an enzyme-linked antibody against BrdU followed, which allowed photometrical quantification of incorporated BrdU at 450 nm.

### Clonogenicity assays

500 cells were seeded into 6-well culture dishes and incubated for seven days (14 days for cell lines ACHN and A704) prior to crystal violet staining and colony counting with ClonoCounter [30].

### *Ex vivo* tissue slice technique

Fresh human renal cell carcinoma tissue samples were obtained from the Tissue Bank of the Center for National Tumor Diseases (NCT, Heidelberg, Germany) directly after surgery. They were maintained in DMEM medium on ice, cut into 300 μm thick slices (Leica VT1200 S vibrating blade microtome; Leica, Wetzlar, Germany). Tissue slices were then placed onto porous filter membranes, suspended in six-well plates and cultured in DMEM supplemented with penicillin (100 U/ml, Sigma Aldrich, St. Louis, MO, USA) and steptomycin (100 mg/ml, Sigma Aldrich) in a conventional CO_2_ incubator. Slices were then incubated in 10 μM Everolimus for 72 h. Approvement of the local ethics committee of the University Hospital of Heidelberg, Germany, was acquired for usage of tumor tissue for research purposes. Anonymization of the data was performed prior to analysis of the data and written consent from the donors or the next of kin obtained for the use of these samples in research.

### ^35^S-methionine labeling

Protein synthesis was assessed through labeling of cells with the beta-emitting radioisotope ^35^S-methionine. Cells were treated with 1 μM Everolimus in Methionine depleted RMPI medium, followed by addition of radioactive ^35^S-methionine for one hour. They were then lysed in 0.2N NaOH, part of which was used for Lowry protein concentration measurement [31]. BSA and 50 % trichloracetic acid was added, the sample 10 min incubated on ice followed by centrifugation at 15 000 rpm for 5 min. After addition of 0.25N NaOH, 1.25 N trichloracetatacid and ULTIMA GoldTM the sample was measured in a scintillation counter (Packard TriCarb 2900, Meriden, IL). Tissue samples were incubated in 10 μM Everolimus in standard RPMI for 72h and labeled with radioactive ^35^S-methionine for 2 h.

### Patients

Tissue samples from 838 patients with primary renal cell carcinoma (RCC) treated at the Department of Urology at the University of Heidelberg between 1987 and 2005 were collected. The human tissue samples were provided by the Tumor Tissue Bank of the NCT Heidelberg after approval by the ethics committee of the University of Heidelberg (ethics approval number: 206 / 2005). Clinical follow-up was available for all cases. Patients were prospectively evaluated every 3 months for the first 2 years after treatment, every 6 months for the next 3 years, and yearly thereafter (chest x-ray or thoracic CT scan; abdominal sonography or CT scan or MRI; serum chemistry). Survival was calculated from the date of nephrectomy until last visit or death. Follow-up was performed according to the guidelines. All tissue samples were reviewed by experienced pathologists. The tumors were graded according to the 4-tiered nuclear grading system [32] and pathologically staged based on the TNM classification (2002). A tissue micro array containing 838 primary and corresponding normal tissue samples was created. All cases of RCC were centrally reviewed by an experienced GU pathologist (S.M.G.). Sections were cut from representative donor blocks for the tissue microarray, and stained with hematoxylin and eosin. Afterwards, morphologically representative regions were chosen from the tumor and normal renal tissue samples. With the help of a semiautomatic system (Beecher Instruments, Silver Spring, MD), two cyclindrical core tissue specimens were punched from these regions and arrayed into a recipient paraffin block. 19 tissue arrays were generated, each containing 200 core tissue samples, according to 50 patients per array [14, 15].

### Immunohistochemistry

The tissue micro array slides were dewaxed and rehydrated using xylene and a series of graded alcohols, followed by heat-induced antigen retrieval using a target retrieval solution (S2031, DakoCytomation, Glostrup, Denmark) in a pressure cooker for 10 min. Staining was performed using an automated staining system (Techmate 500, DakoCytomation) with anti-rpS6 (S6 (5G10) Rabbit mAb #2217, Cell Signaling, Boston, USA) and anti-p-rpS6 (S6-p240 Mouse mAbDAK-S6-240, DakoCytomation, Glostrup, Denmark) for 90 min, and avidin-biotin-complex peroxidase technique using aminoethylcarbazole for visualisation and hematoxylin for counterstaining. In accordance with the manufacturers' instructions, the following solutions were used: ChemMate Detection Kit (K5003, DakoCytomation, containing Dako REAL™ Link, ready-to-use biotinylated goat anti-mouse and anti-rabbit immunoglobulins, and Dako REAL™ AEC/H2O2 Substrate Solution), ChemMate Buffer Kit (K5006, DakoCytomation), and for reduction of non-specific avidin/biotin-related staining Avidin/Biotin Blocking Kit (SP-2001, Vector Laboratories, Burlingame, U.S.A.). As a negative control for the immunohistochemical staining procedure, the primary antibody was omitted or an isotype control antibody (IgG1) was used, with all other experimental conditions kept constant. For the immunohistochemical assessment of rpS6 and p-rpS6 expression, intensity was divided into four groups: 0 = negative to very low, 1 = low, 2 = medium, 3 = high expression. The arrays were independently scored by two pathologists (MK, SMG) blinded to tissue annotations and patient outcomes. In the few instances of discrepant scoring, a consensus score was determined.

### Statistical methods

Data were analysed using the R software package (version 3.0.1, http://www.rproject.org). For count data, Fisher's exact test (two-sided) was used. The Kaplan-Meier method was applied to calculate survival rates for both progression-free and cancer-specific overall survival. For multivariate analysis, the Cox proportional hazards regression model was used. Univariate survival data were tested for significance using the Mantel-Haenszel log rank test. P-values less than 0.05 were considered significant.

## SUPPLEMENTARY MATERIAL FIGURES AND TABLES


