# Supplementary material for: The ribosomal protein S6 in renal cell carcinoma: functional relevance and potential as biomarker
Source: Oncotarget. 2015 Oct 25;7(1):418–32. doi: 10.18632/oncotarget.6225 (PMC4808008; doi:10.18632/oncotarget.6225)
Supplement: Supplementary file 1 [file oncotarget-07-0418-s001.pdf]

The ribosomal protein S6 in renal cell carcinoma: functional relevance and potential as biomarker

Supplementary Material

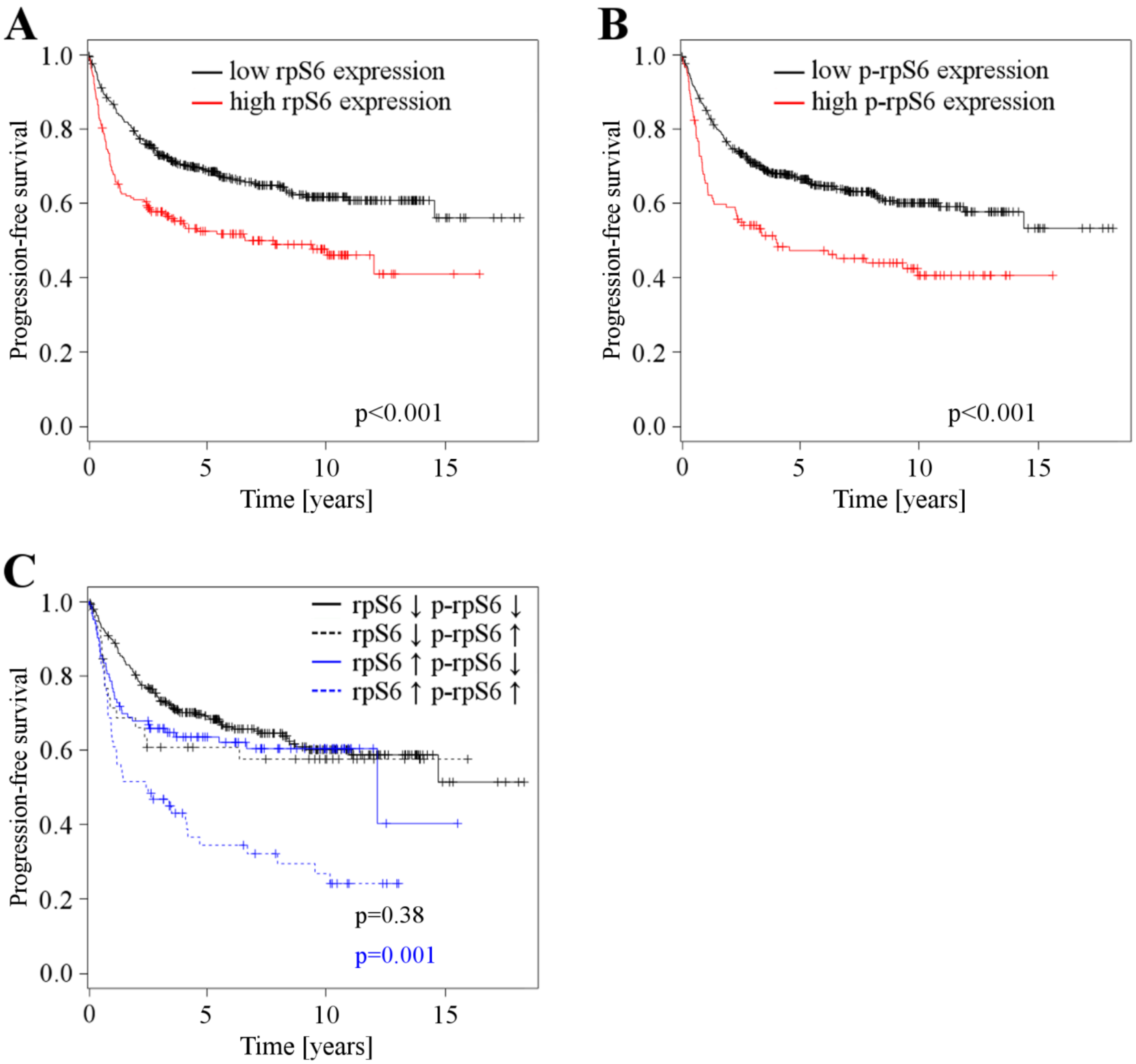

Supplementary Figure 1 A: Progression free survival depending on rpS6 expression levels (n=580), patients with low rpS6 expression levels (n=387) vs. patients with high rpS6 expression levels (n=193). B: Progression free survival depending on p-rpS6 expression levels (n=598), patients with low p-rpS6 expression levels (n=473) vs. patients with high p-rpS6 expression levels (n=125). C: Progression free survival depending on p-rpS6 expression in patients with low (n=339) and high rpS6 (n=167) expression. Patients with low rpS6 expressing RCCs are further divided into groups with low (n=300) and high (n=39) p-rpS6 expression, the same partitioning is done for patients with high rpS6 expression: low p-rpS6 (n=103) and high p-rpS6 (n=64).

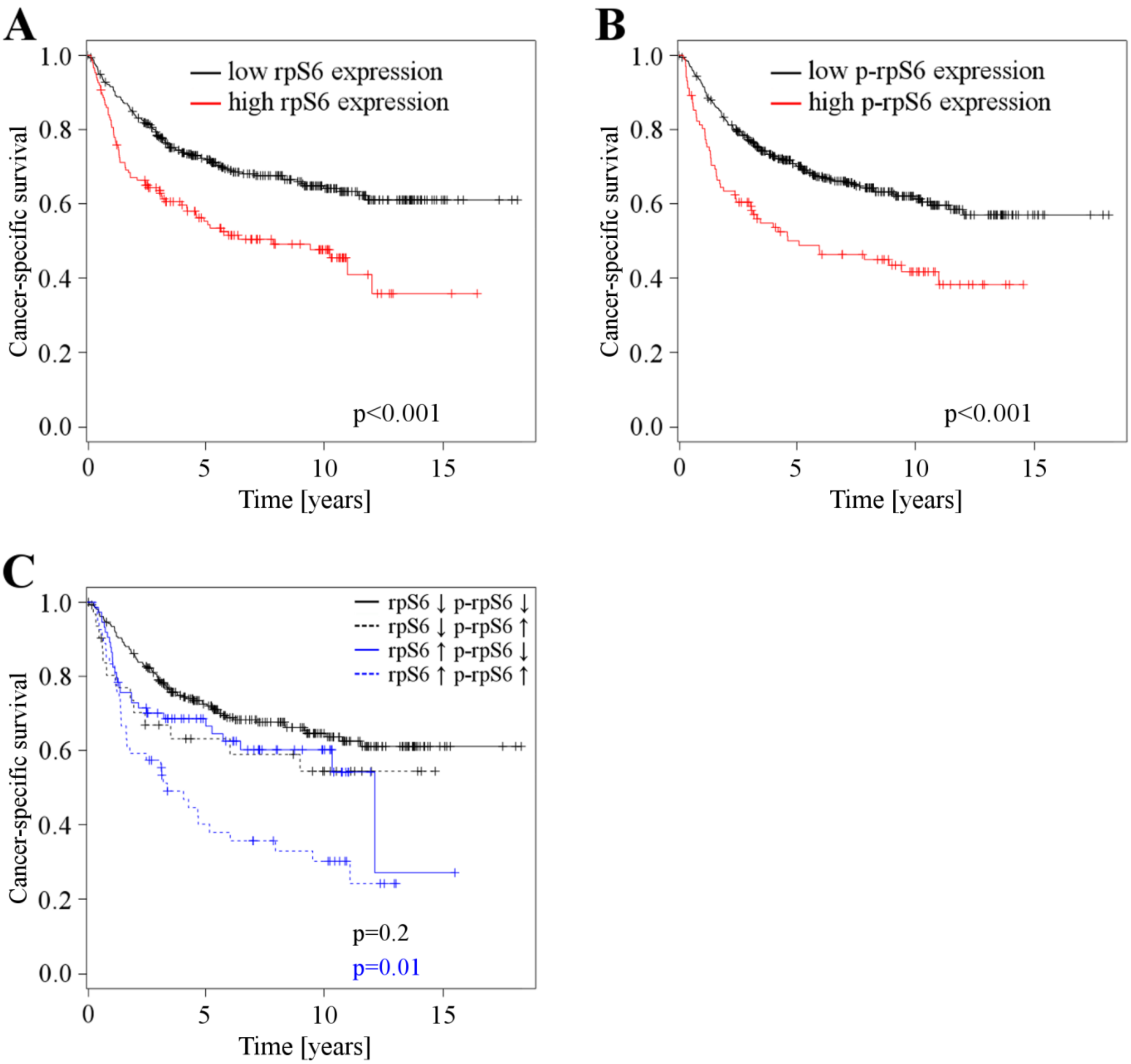

Supplementary Figure 2 A: Cancer specific survival in patients with ccRCC depending on rpS6 expression levels (n=486), patients with low rpS6 expression levels (n=336) vs. patients with high rpS6 expression levels (n=150). B: Cancer specific survival in patients with ccRCC depending on p-rpS6 expression levels (n=496), patients with low p-rpS6 expression levels (n=394) vs. patients with high p-rpS6 expression levels (n=102). C: Cancer specific survival in patients with ccRCC depending on p-rpS6 expression in patients with low (n=294) and high rpS6 (n=128) expression. Patients with low rpS6 expressing RCCs are further divided into groups with low (n=263) and high (n=31) p-rpS6 expression, the same partitioning is done for patients with high rpS6 expression: low p-rpS6 (n=74) and high p-rpS6 (n=54).

Suppl. Tab. 1: **Comparison of rpS6 / p-rpS6 expression levels and clinical and pathologic features in patients with localized (M0) and metastasized (M1) RCC.**

	M0						M1					
Feature	**rpS6**			**p-rpS6**			**rpS6**			**p-rpS6**		
	high	low	p	high	low	p	high	low	p	high	low	p
	147 (32)	319 (68)		92 (19)	394 (81)		46 (40)	68 (60)		33 (29)	79 (71)	
**Sex**			1			0.723			1			0.259
Male	86 (18)	186 (40)		57 (12)	234 (48)		32 (28)	47 (41)		26 (23)	52 (46)	
Female	61 (13)	133 (29)		35 (7)	160 (33)		14 (12)	21 (18)		7 (6)	27 (24)	
**Age at surgery (y)**			0.761			0.814			0.693			0.286
<65	86 (18)	192 (41)		57 (12)	238 (49)		31 (27)	43 (38)		19 (17)	54 (48)	
>=65	61 (13)	127 (27)		35 (7)	156 (32)		15 (13)	25 (22)		14 (12)	25 (22)	
**Karnofsky index**			**0.02**			**0.001**			0.447			0.836
>=1	52 (11)	79 (17)		41 (8)	102 (21)		22 (19)	38 (33)		19 (17)	43 (38)	
<1	95 (20)	240 (52)		51 (10)	292 (60)		24 (21)	30 (26)		14 (12)	36 (32)	
**Tumor extent**			0.825			0.124			**0.024**			0.191
pT1/2	107 (23)	228 (49)		60 (12)	289 (59)		9 (8)	28 (25)		8 (7)	31 (28)	
pT3/4	40 (9)	91 (20)		32 (7)	105 (22)		37 (32)	40 (35)		25 (22)	48 (43)	
**Regional lymph node metastasis**			0.219			**0.006**			0.093			0.154
N0/pN0	138 (30)	308 (66)		83 (17)	383 (79)		29 (25)	53 (46)		21 (19)	62 (55)	
pN1, pN2	9 (2)	11 (2)		9 (2)	11 (2)		17 (15)	15 (13)		12 (11)	17 (15)	
**Grade of malignancy**			0.078			**0.018**			0.124			0.064
G1/2	125 (27)	290 (62)		74 (15)	355 (73)		21 (18)	42 (37)		14 (12)	49 (44)	
G3/4	22 (5)	29 (6)		18 (4)	39 (8)		25 (22)	26 (23)		19 (17)	30 (27)	
**Type of surgery**			**0.003**			1						1
Radical nephrectomy	107 (23)	271 (58)		76 (16)	323 (66)					33 (29)	78 (70)	
Partial nephrectomy	40 (9)	48 (10)		16 (3)	71 (15)					0 (0)	1 (1)	
**Histopathological subtype**			**0.025**			0.655			0.079			0.549
Clear-cell	113 (24)	273 (59)		74 (15)	324 (67)		37 (32)	63 (55)		28 (25)	70 (62)	
Non clear-cell	34 (7)	46 (10)		18 (4)	70 (14)		9 (8)	5 (4)		5 (4)	9 (8)	

Suppl. Tab. 2: **Comparison of rpS6 / p-rpS6 expression levels and clinical and pathologic features in patients with localized (N0) and lymph node-metastasized (N1/2) RCC.**

	M0											
	N1/2						N0					
Feature	**rpS6**			**p-rpS6**			**rpS6**			**p-rpS6**		
	high	low	p	high	low	p	high	low	p	high	low	p
	9 (45)	11 (55)		9 (45)	11 (55)		138 (31)	308 (69)		83 (18)	383 (82)	
**Sex**			1			0.617			1			0.712
Male	6 (30)	8 (40)		6 (30)	9 (45)		80 (18)	178 (40)		51 (11)	225 (48)	
Female	3 (15)	3 (15)		3 (15)	2 (10)		58 (13)	130 (29)		32 (7)	158 (34)	
**Age at surgery (y)**			0.374			0.406			1			0.459
<65	2 (10)	5 (25)		3 (15)	6 (30)		84 (19)	187 (42)		54 (12)	232 (50)	
>=65	7 (35)	6 (30)		6 (30)	5 (25)		54 (12)	121 (27)		29 (6)	151 (32)	
**Karnofsky index**			0.175			0.362			0.066			**0.002**
>=1	6 (30)	3 (15)		5 (25)	3 (15)		46 (10)	76 (17)		36 (8)	99 (21)	
<1	3 (15)	8 (40)		4 (20)	8 (40)		92 (21)	232 (52)		47 (10)	284 (61)	
**Tumor extent**			1			0.285			0.561			0.217
pT1/2	2 (10)	2 (10)		3 (15)	1 (5)		105 (24)	226 (51)		57 (12)	288 (62)	
pT3/4	7 (35)	9 (45)		6 (30)	10 (50)		33 (7)	82 (18)		26 (6)	95 (20)	
**Grade of malignancy**			0.642			1			0.168			**0.031**
G1/2	5 (25)	8 (40)		6 (30)	8 (40)		120 (27)	282 (63)		68 (15)	347 (74)	
G3/4	4 (20)	3 (15)		3 (15)	3 (15)		18 (4)	26 (6)		15 (3)	36 (8)	
**Type of surgery**			1			0.45			**0.001**			1
Radical nephrectomy	9 (45)	10 (50)		8 (40)	11 (55)		98 (22)	261 (59)		68 (15)	312 (67)	
Partial nephrectomy	0 (0)	1 (5)		1 (5)	0 (0)		40 (9)	47 (11)		15 (3)	71 (15)	
**Histopathological subtype**			0.566			0.566			**0.03**			0.756
Clear-cell	7 (35)	10 (50)		7 (35)	10 (50)		106 (24)	263 (59)		67 (14)	314 (67)	
Non clear-cell	2 (10)	1 (5)		2 (10)	1 (5)		32 (7)	45 (10)		16 (3)	69 (15)	

Suppl. Tab. 3: Uni- and multivariate analyses of rpS6 and p-rpS6 expression and clinical/pathologic features for the prediction of progression free survival in patients with RCCs.

	**M0+M1**				**M0**				**M1**			
	Univariate Analysis Hazard Ratio (95% CI)	p	Multivariate Analysis Hazard Ratio (95% CI)	p	Univariate Analysis Hazard Ratio (95% CI)	p	Multivariate Analysis Hazard Ratio (95%	p	Univariate Analysis Hazard Ratio (95% CI)	p	Multivariate Analysis Hazard Ratio (95% CI)	p
**rpS6 + p-rps6 Expression∗**	1.7 (1.3-2.2)	**<0.001**	1.8 (1.3-2.4)	**<0.001**	1.8 (1.2-2.5)	**0.003**	1.7 (1.1-2.6)	**0.013**	2.4 (1.5-3.7)	**<0.001**	2.4 (1.6-3.9)	**<0.001**
Karnofsky*	1.9 (1.4-2.5)	**<0.001**	1.3 (0.9-1.7)	0.128	1.4 (0.9-2)	0.097	1.3 (0.9-1.9)	0.244	1.2 (0.8-1.9)	0.378	1.4 (0.9-2.2)	0.17
Tumor extent◦	2.4 (1.8-3.2)	**<0.001**	1.4 (1-1.9)	**0.032**	1.3 (0.9-1.9)	0.175	1.4 (0.9-2.1)	0.141	1.8 (1.1-2.8)	**0.02**	1.5 (0.9-2.5)	0.122
Regional lymph node metastasis•	2.7 (1.9-3.9)	**<0.001**	1.2 (0.8-1.7)	0.386	1.3 (0.8-2.3)	0.335	1.2 (0.7-2.1)	0.574	1.5 (0.9-2.3)	0.115	0.8 (0.5-1.4)	0.395
Distant metastasis[±]	77.4 (41.3-	**<0.001**	80.5 (40.9-	**<0.001**								
Grade of malignancy⊗	2.7 (2-3.6)	**<0.001**	1.3 (0.9-1.8)	0.11	1.4 (0.9-2.1)	0.15	1 (0.6-1.6)	0.975	1.9 (1.2-2.9)	**0.003**	1.8 (1.2-2.9)	**0.009**
Type of surgery t	2 (1.1-3.7)	**0.028**	0.5 (0.3-1)	0.053	0.7 (0.4-1.3)	0.265	0.6 (0.3-1.1)	0.113				
Sex=	1.3 (1-1.8)	0.053	1 (0.7-1.3)	0.878	1.2 (0.8-1.8)	0.307	1.1 (0.7-1.6)	0.777	0.9 (0.5-1.4)	0.511	0.8 (0.5-1.4)	0.486
Histopathologic subtype<	1.3 (1-1.8)	0.053	0.8 (0.5-1.3)	0.429	1.2 (0.8-1.8)	0.307	1.3 (0.6-2.5)	0.517	0.9 (0.5-1.4)	0.511	0.4 (0.2-0.8)	**0.011**
**rpS6 Expression∗**	1.7 (1.3-2.2)	**<0.001**	1.6 (1.2-2.2)	**0.001**	1.7 (1.2-2.4)	**0.005**	1.5 (1-2.3)	**0.039**	2.3 (1.5-3.4)	**<0.001**	2.1 (1.4-3.2)	**0.001**
Karnofsky*	1.8 (1.4-2.4)	**<0.001**	1.3 (1-1.8)	**0.036**	1.3 (0.9-1.9)	0.101	1.3 (0.9-1.9)	0.208	1.3 (0.9-2)	0.157	1.6 (1.1-2.5)	**0.029**
Tumor extent◦	2.5 (1.9-3.3)	**<0.001**	1.4 (1-1.9)	**0.031**	1.4 (1-2)	0.079	1.4 (0.9-2.1)	0.12	1.8 (1.2-2.8)	**0.009**	1.4 (0.9-2.3)	0.169
Regional lymph node metastasis•	2.8 (2-3.8)	**<0.001**	1.2 (0.8-1.6)	0.4	1.5 (0.9-2.4)	0.129	1.5 (0.9-2.5)	0.132	1.2 (0.8-1.9)	0.385	0.8 (0.5-1.4)	0.489
Distant metastasis[±]	61.5 (35.7-	**<0.001**	57.9 (32.2-	**<0.001**								
Grade of malignancy⊗	2.9 (2.2-3.9)	**<0.001**	1.4 (1-1.8)	**0.048**	1.6 (1-2.3)	**0.03**	1.2 (0.7-1.9)	0.517	1.8 (1.2-2.7)	**0.003**	1.7 (1.1-2.6)	**0.012**
Type of surgery t	2.3 (1.3-4)	**0.007**	0.6 (0.3-1)	0.066	0.7 (0.4-1.3)	0.322	0.6 (0.3-1.1)	0.088				
Sex=	1.4 (1-1.8)	**0.024**	1.1 (0.8-1.5)	0.521	1.3 (0.9-1.8)	0.24	1.1 (0.7-1.6)	0.722	1 (0.6-1.5)	0.98	1 (0.7-1.6)	0.956
Histopathologic subtype<	1.4 (1-1.8)	**0.024**	0.8 (0.5-1.2)	0.284	1.3 (0.9-1.8)	0.24	1 (0.6-1.9)	0.939	1 (0.6-1.5)	0.98	0.5 (0.3-0.9)	**0.03**
**p-rpS6 Expression∗**	1.5 (1.1-2)	**0.006**	1.2 (0.9-1.6)	0.352	1.2 (0.8-1.8)	0.268	1 (0.7-1.5)	0.965	1.6 (1.1-2.5)	**0.025**	1.6 (1-2.5)	**0.032**
Karnofsky*	1.9 (1.4-2.4)	**<0.001**	1.4 (1.1-1.8)	**0.018**	1.4 (1-1.9)	0.067	1.4 (1-2)	0.076	1.2 (0.8-1.8)	0.289	1.5 (1-2.3)	0.063
Tumor extent◦	2.4 (1.8-3.1)	**<0.001**	1.4 (1-1.8)	**0.043**	1.4 (1-1.9)	0.075	1.4 (0.9-2)	0.127	1.7 (1.1-2.5)	**0.023**	1.4 (0.9-2.2)	0.168
Regional lymph node metastasis•	2.5 (1.8-3.4)	**<0.001**	1.2 (0.8-1.7)	0.318	1.2 (0.8-2)	0.38	1.2 (0.7-2.1)	0.417	1.4 (0.9-2.2)	0.107	0.9 (0.5-1.4)	0.55
Distant metastasis[±]	56.8 (34.2-	**<0.001**	51.6 (30.1-									
Grade of malignancy⊗	2.7 (2.1-3.5)	**<0.001**	1.5 (1.2-2.1)	**0.003**	1.5 (1-2.2)	0.055	1.4 (0.9-2.1)	0.16	2 (1.3-2.9)	**0.001**	2 (1.3-3)	**0.001**
Type of surgery t	1.7 (1-2.8)	**0.046**	0.5 (0.3-0.8)	**0.009**	0.6 (0.4-1.1)	0.087	0.5 (0.3-0.8)	**0.01**	1.8 (0.3-	0.546	0.8 (0.1-5.8)	0.792
Sex=	1.4 (1-1.8)	**0.029**	1 (0.8-1.4)	0.761	1.3 (0.9-1.9)	0.155	1.2 (0.8-1.7)	0.424	0.9 (0.6-1.3)	0.554	0.9 (0.6-1.4)	0.572
Histopathologic subtype<	1.4 (1-1.8)	**0.029**	0.8 (0.5-1.2)	0.2	1.3 (0.9-1.9)	0.155	1 (0.6-1.8)	0.961	0.9 (0.6-1.3)	0.554	0.4 (0.2-0.8)	**0.008**

Suppl. Tab. 4: Uni- and multivariate analyses of rpS6 and p-rpS6 expression and clinical/pathologic features for the prediction of cancer specific survival in patient with localized or metastasized RCCs.

	M0				M1			
	Univariate Analysis		Multivariate Analysis		Univariate Analysis		Multivariate Analysis	
	Hazard Ratio (95% CI)	p	Hazard Ratio (95% CI)	p	Hazard Ratio (95% CI)	p	Hazard Ratio (95% CI)	p
**rpS6 + p-rps6 Expression⁎**	1.7 (1.1-2.6)	**0.009**	1.9 (1.2-2.9)	**0.007**	2.4 (1.5-3.7)	**<0.001**	2.4 (1.6-3.9)	**<0.001**
Karnofsky*	2.4 (1.6-3.6)	**<0.001**	1.9 (1.2-2.9)	**0.003**	1.2 (0.8-1.9)	0.378	1.4 (0.9-2.2)	0.17
Tumor extent◦	6.1 (4-9.3)	**<0.001**	4.6 (2.8-7.3)	**<0.001**	1.8 (1.1-2.8)	**0.02**	1.5 (0.9-2.5)	0.122
Regional lymph node metastasis•	9.9 (5.6-17.3)	**<0.001**	5.2 (2.9-9.2)	**<0.001**	1.5 (0.9-2.3)	0.115	0.8 (0.5-1.4)	0.395
Grade of malignancy⊗	4.9 (3.1-7.8)	**<0.001**	1.7 (1-2.9)	**0.042**	1.9 (1.2-2.9)	**0.003**	1.8 (1.2-2.9)	**0.009**
Type of surgery t	2.5 (1.2-5.4)	**0.019**	1.5 (0.7-3.3)	0.344				
Sex=	1.7 (1.1-2.7)	**0.012**	1.5 (1-2.4)	0.065	0.9 (0.5-1.4)	0.511	0.8 (0.5-1.4)	0.486
Histopathologic subtype<	1.7 (1.1-2.7)	**0.012**	1.9 (0.9-4.2)	0.107	0.9 (0.5-1.4)	0.511	0.4 (0.2-0.8)	**0.011**
**rpS6 Expression⁎**	1.8 (1.2-2.7)	**0.003**	1.6 (1.1-2.5)	**0.028**	2.3 (1.5-3.4)	**<0.001**	2.1 (1.4-3.2)	**0.001**
Karnofsky*	2.2 (1.5-3.3)	**<0.001**	1.7 (1.1-2.6)	**0.009**	1.3 (0.9-2)	0.157	1.6 (1.1-2.5)	**0.029**
Tumor extent◦	6.3 (4.2-9.4)	**<0.001**	3.9 (2.5-6.2)	**<0.001**	1.8 (1.2-2.8)	**0.009**	1.4 (0.9-2.3)	0.169
Regional lymph node metastasis•	8.5 (5-14.4)	**<0.001**	4 (2.3-6.9)	**<0.001**	1.2 (0.8-1.9)	0.385	0.8 (0.5-1.4)	0.489
Grade of malignancy⊗	5.7 (3.7-8.7)	**<0.001**	2.3 (1.4-3.8)	**0.001**	1.8 (1.2-2.7)	**0.003**	1.7 (1.1-2.6)	**0.012**
Type of surgery t	2.7 (1.3-5.5)	**0.007**	1.3 (0.6-2.8)	0.509				
Sex=	1.7 (1.1-2.6)	**0.01**	1.7 (1.1-2.6)	**0.018**	1 (0.6-1.5)	0.98	1 (0.7-1.6)	0.956
Histopathologic subtype<	1.7 (1.1-2.6)	**0.01**	1.8 (0.9-3.8)	0.103	1 (0.6-1.5)	0.98	0.5 (0.3-0.9)	**0.03**
**p-rpS6 Expression⁎**	1.9 (1.2-2.8)	**0.003**	1.4 (0.9-2.1)	0.152	1.6 (1.1-2.5)	**0.025**	1.6 (1-2.5)	**0.032**
Karnofsky*	2 (1.4-3)	**<0.001**	1.9 (1.3-2.8)	**0.001**	1.2 (0.8-1.8)	0.289	1.5 (1-2.3)	0.063
Tumor extent◦	6.2 (4.2-9.2)	**<0.001**	4.3 (2.8-6.7)	**<0.001**	1.7 (1.1-2.5)	**0.023**	1.4 (0.9-2.2)	0.168
Regional lymph node metastasis•	9.5 (5.8-15.7)	**<0.001**	4.8 (2.9-8.2)	**<0.001**	1.4 (0.9-2.2)	0.107	0.9 (0.5-1.4)	0.55
Grade of malignancy⊗	4.8 (3.2-7.2)	**<0.001**	1.9 (1.2-3.1)	**0.005**	2 (1.3-2.9)	**0.001**	2 (1.3-3)	**0.001**
Type of surgery t	2 (1.1-3.8)	**0.026**	1 (0.5-1.9)	0.91	1.8 (0.3-13.2)	0.546	0.8 (0.1-5.8)	0.792
Sex=	1.9 (1.2-2.8)	**0.003**	1.7 (1.1-2.6)	**0.013**	0.9 (0.6-1.3)	0.554	0.9 (0.6-1.4)	0.572
Histopathologic subtype<	1.9 (1.2-2.8)	**0.003**	1.7 (0.9-3.3)	0.106	0.9 (0.6-1.3)	0.554	0.4 (0.2-0.8)	**0.008**

⁎Immunhistochemical intensity high vs. low

* <80% vs >= 80%

◦pT3/pT4 vs pT1/pT2

• pN1/pN2 vs pN0/N0

± M1 vs M0

⊗G3/G4 vs G1/G2

= Male vs female

t Radical vs partial nephrectomy

< Clear-cell vs non-clear-cell

Suppl. Tab. 5: **Comparison of rpS6 / p-rpS6 expression levels and clinical and pathologic features in patients with ccRCC.**

	M0+M1						M0						M1					
	**rps6**			**p-rpS6**			**rps6**			**p-rps6**			**rps6**			**p-rps6**		
	high	low	p	high	low	p	high	low	p	high	low	p	high	low	p	high	low	p
	150 (31)	336 (69)		102 (21)	394 (79)		113	273 (71)		74 (19)	324 (81)		37 (37)	63 (63)		28 (29)	70 (71)	
**Sex**			0.921			0.57			0.822			1			1			0.458
Male	89 (18)	201 (41)		65 (13)	238 (48)		63 (16)	156 (40)		43 (11)	190 (48)		26 (26)	45 (45)		22 (22)	48 (49)	
Female	61 (13)	135 (28)		37 (7)	156 (31)		50 (13)	117 (30)		31 (8)	134 (34)		11 (11)	18 (18)		6 (6)	22 (22)	
**Age at surgery (y)**			1			0.65			0.57			0.895			0.39			0.645
<65	91 (19)	204 (42)		60 (12)	242 (49)		65 (17)	166 (43)		44 (11)	197 (49)		26 (26)	38 (38)		16 (16)	45 (46)	
>=65	59 (12)	132 (27)		42 (8)	152 (31)		48 (12)	107 (28)		30 (8)	127 (32)		11 (11)	25 (25)		12 (12)	25 (26)	
**Karnofsky index**			**0.023**			**<0.001**			**0.014**			**<0.001**			0.83			0.65
>=1	63 (13)	105 (22)		55 (11)	123 (25)		43 (11)	69 (18)		37 (9)	83 (21)		20 (20)	36 (36)		18 (18)	40 (41)	
<1	87 (18)	231 (48)		47 (9)	271 (55)		70 (18)	204 (53)		37 (9)	241 (61)		17 (17)	27 (27)		10 (10)	30 (31)	
**Tumor extent**			0.055			**0.011**			0.547			0.162			**0.05**			0.113
pT1/2	84 (17)	219 (45)		53 (11)	260 (52)		76 (20)	192 (50)		46 (12)	230 (58)		8 (8)	27 (27)		7 (7)	30 (31)	
pT3/4	66 (14)	117 (24)		49 (10)	134 (27)		37 (10)	81 (21)		28 (7)	94 (24)		29 (29)	36 (36)		21 (21)	40 (41)	
**Regional lymph**			**0.021**			**0.002**			0.282			**0.023**			0.13			0.165
N0/pN0	131 (27)	315 (65)		87 (18)	373 (75)		106	263 (68)		67 (17)	314 (79)		25 (25)	52 (52)		20 (20)	59 (60)	
pN1, pN2	19 (4)	21 (4)		15 (3)	21 (4)		7 (2)	10 (3)		7 (2)	10 (3)		12 (12)	11 (11)		8 (8)	11 (11)	
**Distant metastasis**			0.146			**0.036**												
M0	113 (23)	273 (56)		74 (15)	324 (65)													
M1	37 (8)	63 (13)		28 (6)	70 (14)													
**Grade of malignancy**			**0.007**			**0.001**			**0.037**			**0.006**			0.40			0.263
G1/2	112 (23)	286 (59)		71 (14)	334 (67)		93 (24)	247 (64)		57 (14)	290 (73)		19 (19)	39 (39)		14 (14)	44 (45)	
G3/4	38 (8)	50 (10)		31 (6)	60 (12)		20 (5)	26 (7)		17 (4)	34 (9)		18 (18)	24 (24)		14 (14)	26 (27)	
**Type of surgery**			**0.003**			1			**0.001**			0.734						1
Radical	118 (24)	300 (62)		88 (18)	338 (68)		81 (21)	237 (61)		60 (15)	269 (68)					28 (29)	69 (70)	
Partial	32 (7)	36 (7)		14 (3)	56 (11)		32 (8)	36 (9)		14 (4)	55 (14)					0 (0)	1 (1)	

Suppl. Tab. 6: Uni- and multivariate analyses of rpS6 and p-rpS6 expression and clinical/pathologic features for the prediction of cancer specific survival in patients with ccRCCs.

	M0+M1				M0				M1			
	Univariate Analysis		Multivariate Analysis		Univariate Analysis		Multivariate Analysis		Univariate Analysis		Multivariate Analysis	
	Hazard Ratio (95% CI)	p	Hazard Ratio (95% CI)	p	Hazard Ratio (95% CI)	p	Hazard Ratio (95% CI)	p	Hazard Ratio (95% CI)	p	Hazard Ratio (95% CI)	p
**rpS6 + p-rps6**	1.8 (1.3-2.5)	**<0.001**	1.8 (1.3-2.5)	**<0.001**	1.9 (1.2-2.9)	**0.003**	1.7 (1-2.7)	**0.033**	2.2 (1.4-3.4)	**0.001**	2.3 (1.4-3.7)	**0.001**
Karnofsky*	2.6 (1.9-3.5)	**<0.001**	1.6 (1.2-2.3)	**0.003**	2.2 (1.4-3.3)	**<0.001**	1.9 (1.2-2.9)	**0.007**	1.3 (0.8-2.1)	0.253	1.3 (0.8-2.1)	0.346
Tumor extent◦	5.1 (3.7-7.1)	**<0.001**	2.9 (2-4.2)	**<0.001**	6 (3.8-9.3)	**<0.001**	4.8 (2.9-7.9)	**<0.001**	1.7 (1.1-2.8)	**0.03**	1.6 (0.9-2.7)	0.103
Regional lymph node metastasis•	5.7 (3.8-8.5)	**<0.001**	1.5 (1-2.3)	0.072	8.1 (4.5-14.5)	**<0.001**	4.8 (2.6-8.7)	**<0.001**	1.4 (0.8-2.4)	0.219	0.8 (0.4-1.5)	0.517
Distant metastasis ±	8.9 (6.4-12.3)	**<0.001**										
Grade of malignancy ⨂	4.4 (3.2-6.2)	**<0.001**	1.6 (1.1-2.3)	**0.007**	4.3 (2.6-7)	**<0.001**	1.7 (1-2.9)	0.052	1.7 (1.1-2.6)	**0.03**	1.7 (1-2.7)	**0.044**
Type of surgery t	3.6 (1.7-7.7)	0.001	1.6 (0.7-3.5)	0.264	2.1 (1-4.5)	**0.066**	1.2 (0.5-2.7)	0.695				
Sex=	1.8 (1.3-2.5)	0.001	1.3 (1-1.9)	0.095	1.8 (1.1-2.8)	**0.012**	1.4 (0.9-2.3)	0.13	1 (0.6-1.6)	0.913	0.9 (0.5-1.5)	0.635
**rpS6 Expression∗**	1.9 (1.4-2.5)	**<0.001**	1.7 (1.3-2.3)	**0.001**	1.9 (1.2-2.8)	**0.003**	1.4 (0.9-2.2)	0.138	2.1 (1.3-3.2)	**0.001**	2.1 (1.3-3.4)	**0.001**
Karnofsky*	2.4 (1.8-3.3)	**<0.001**	1.7 (1.3-2.3)	**0.001**	2.1 (1.4-3.1)	**<0.001**	1.7 (1.1-2.6)	**0.012**	1.4 (0.9-2.2)	0.119	1.4 (0.9-2.2)	0.121
Tumor extent◦	5.3 (3.9-7.3)	**<0.001**	2.9 (2-4.1)	**<0.001**	6 (3.9-9.2)	**<0.001**	4.2 (2.6-6.7)	**<0.001**	1.9 (1.2-3)	**0.01**	1.6 (1-2.7)	0.062
Regional lymph node metastasis•	5 (3.4-7.2)	**<0.001**	1.2 (0.8-1.9)	0.314	7.2 (4.1-12.6)	**<0.001**	3.7 (2.1-6.5)	**<0.001**	1.1 (0.7-1.9)	0.597	0.7 (0.4-1.3)	0.294
Distant metastasis ±	8.7 (6.4-11.9)	**<0.001**	4.6 (3.2-6.5)	**<0.001**								
Grade of malignancy ⨂	4.9 (3.6-6.6)	**<0.001**	1.8 (1.3-2.5)	**0.001**	4.9 (3.1-7.7)	**<0.001**	2.3 (1.4-3.8)	**0.002**	1.7 (1.1-2.6)	**0.017**	1.7 (1.1-2.7)	**0.02**
Type of surgery t	4 (2-8.1)	**<0.001**	1.5 (0.7-3.3)	0.254	2.3 (1.1-4.7)	**0.027**	1.1 (0.5-2.3)	0.875				
Sex=	1.9 (1.3-2.5)	**<0.001**	1.6 (1.1-2.2)	**0.009**	1.9 (1.2-2.9)	**0.005**	1.7 (1.1-2.6)	**0.027**	1.1 (0.7-1.8)	0.667	1.1 (0.7-1.8)	0.73
**p-rpS6 Expression∗**	1.9 (1.4-2.6)	**<0.001**	1.4 (1-1.9)	0.052	1.9 (1.2-2.9)	**0.004**	1.3 (0.8-2.1)	0.228	1.6 (1-2.4)	0.059	1.6 (1-2.6)	0.066
Karnofsky*	2.3 (1.8-3.1)	**<0.001**	1.7 (1.2-2.2)	**0.001**	1.9 (1.3-2.8)	**0.001**	1.8 (1.2-2.8)	**0.005**	1.3 (0.9-2.1)	0.181	1.4 (0.9-2.2)	0.133
Tumor extent◦	4.9 (3.7-6.7)	**<0.001**	2.7 (1.9-3.8)	**<0.001**	5.7 (3.8-8.6)	**<0.001**	4.4 (2.7-7)	**<0.001**	1.6 (1-2.6)	**0.035**	1.4 (0.9-2.3)	0.173
Regional lymph node metastasis•	5.5 (3.8-7.9)	**<0.001**	1.6 (1-2.4)	**0.038**	7.5 (4.4-12.9)	**<0.001**	4.2 (2.4-7.3)	**<0.001**	1.4 (0.8-2.3)	0.234	0.9 (0.5-1.7)	0.767
Distant metastasis ±	9.3 (6.9-12.6)	**<0.001**	4.8 (3.4-6.8)	**<0.001**								
Grade of malignancy ⨂	4.5 (3.3-6)	**<0.001**	1.8 (1.3-2.5)	**<0.001**	4.3 (2.8-6.6)	**<0.001**	1.9 (1.2-3.1)	**0.009**	1.7 (1.1-2.6)	**0.011**	1.7 (1.1-2.7)	**0.017**
Type of surgery t	2.8 (1.6-5.1)	**<0.001**	1 (0.6-1.9)	0.888	1.8 (0.9-3.3)	0.079	0.8 (0.4-1.6)	0.523	1.8 (0.2-	0.575	0.8 (0.1-6.5)	0.872
Sex=	1.8 (1.3-2.5)	**<0.001**	1.5 (1.1-2)	**0.021**	2 (1.3-3)	**0.002**	1.7 (1.1-2.6)	**0.017**	1 (0.6-1.5)	0.869	0.9 (0.6-1.5)	0.805
